# Vitrification-warming delays preimplantation development and impairs mitochondrial function and cytoplasmic lattices integrity in mouse embryos

**DOI:** 10.1016/j.isci.2026.115859

**Published:** 2026-04-22

**Authors:** Mariana T. Barroso, Jose A. Rodriguez Muñoz, Viola Sjöström, Konstantina Dindini, Jian Zhao, Kenny A. Rodriguez-Wallberg, Arturo Reyes Palomares

**Affiliations:** 1 Laboratory of Translational Fertility Preservation, Department of Oncology-Pathology, Karolinska Institutet, Stockholm, Sweden; 2 Gynecology and Reproduction Unit, Department of Reproductive Medicine, Karolinska University Hospital, Stockholm, Sweden; 3 Department of Surgical Specialties, Biochemistry and Immunology, Faculty of Medicine, University of Málaga, Málaga, Spain; 4 IBIMA (Biomedical Research Institute of Málaga and Nanomedicine Platform) - BIONAD Platform, University of Málaga, Andalucia Tech, Málaga, Spain

**Keywords:** Cell biology, Cryopreservation, Developmental biology

## Abstract

Vitrification and warming of human embryos have become standard procedures in assisted reproduction over the past two decades. Although generally considered safe, their full impact on embryo development remains unclear. Epidemiological studies have raised concerns about differences in birth weight and long-term health outcomes between newborns from fresh versus frozen embryo transfers. Here, we present a descriptive study using mouse embryos to investigate the impact of vitrification and warming on developmental kinetics, mitochondrial function, and cytoplasmic lattice integrity. Time-lapse imaging revealed significant developmental delays across all preimplantation stages in vitrified embryos. Additionally, mitochondrial distribution, volume, and membrane potential exhibited signs of impairment. Ultrastructural analysis identified ruptured mitochondrial membranes, disrupted cytoplasmic lattices during early cell divisions, and underdeveloped mitochondrial cristae at the blastocyst stage. We hypothesize that embryo vitrification and warming disrupt mitochondrial function and compromise cytoplasmic lattices integrity, ultimately contributing to developmental delays in preimplantation mouse embryos.

## Highlights

• Vitrification-warming delays development but accelerates compaction before blastocyst
• Mitochondrial ultrastructure and activity are disrupted throughout preimplantation stages
• Ultrastructural analysis reveals reduced cytoplasmic lattices after vitrification-warming
• Vitrification-warming triggers nuclear stress and DNA damage marker activation

## Graphical abstract

**Figure:**
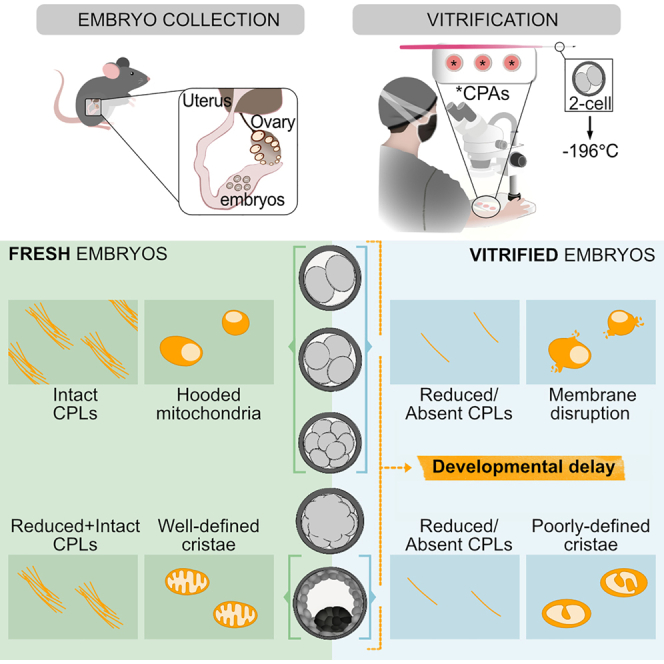

## Introduction

Cryopreservation has revolutionized assisted reproduction, particularly with the implementation of the “freeze-all” policy, which involves storing embryos for future use instead of immediate transfer. This strategy helps mitigate risks of ovarian hyperstimulation and improves success rates in cases involving endometrial anomalies or requiring preimplantation genetic testing.^1^^,^^2^ Additionally, embryo cryopreservation is increasingly used for social freezing, allowing women to preserve their fertility for later childbearing.^3^ According to the European Society of Human Reproduction and Embryology (ESHRE), by 2014, frozen embryo transfers (FETs) accounted for over 50% of all embryo transfers in some European countries.^4^

Despite its widespread adoption, the rapid expansion of cryopreservation procedures has outpaced our ability to fully understand their impact on embryo development and offspring health. Recent population-based registry studies have raised concerns about potential risks associated with embryo cryopreservation, including (1) an increased risk of infant mortality (from birth up to 1 year of life) in singletons resulting from FETs,^5^ (2) an increased risk of obstetrical complications in singleton births following FET of two embryos,^6^ (3) a higher incidence of childhood cancer,^7^^,^^8^ and (4) imprinting disorders in children born from FETs.^9^ A notable strength of these studies is their focus on singleton births, minimizing confounders from multiple pregnancies and allowing clearer comparisons between assisted reproduction and natural conception.

Over the past decade, vitrification has replaced slow freezing as the preferred method for embryo cryopreservation, offering improved success rates for frozen versus fresh embryo transfers.^10^^,^^11^^,^^12^ Slow freezing often led to intracellular ice crystal formation causing cellular damage and reduced survival rates.^13^ In contrast, vitrification prevents ice crystal formation by rapidly cooling embryos to a glass-like state, dramatically improving post-thaw viability.^14^^,^^15^ This is achieved by briefly exposing embryos to high concentrations of cryoprotectant agents (CPAs), followed by immediate submersion into liquid nitrogen for ultrafast cooling.^11^^,^^16^ The Cryotop method, developed by Kuwayama, further refined vitrification by employing a thin carrier that holds embryos in a minimal volume of CPA solution, enabling fast cooling and warming rates.^11^

In clinical practice, embryo vitrification is typically performed at the 4-cell, 8-cell, or blastocyst stage (day 5–6 of development). These stages coincide with a critical period of genome-wide reprogramming in the embryo, including changes in methylation status, chromatin accessibility, degradation of maternal transcripts, and activation of the embryonic genome. While embryos appear morphologically normal after thawing, exposure to non-physiological conditions during this vulnerable period may result in cumulative negative effects.

Studies have shown that CPAs, while essential for vitrification, are also a direct source of toxicity. They have been linked to changes in chromatin architecture, DNA replication fork collapse, and DNA double-strand breaks, all of which contribute to genomic instability.^17^^,^^18^

Furthermore, embryo vitrification has been implicated in altering the expression of imprinting genes by disrupting DNA methylation levels and regulatory regions.^19^^,^^20^^,^^21^^,^^22^^,^^23^^,^^24^ Impairment of these genes can directly influence fetal growth and offspring health,^25^^,^^26^ underscoring the importance of the epigenetic landscape for normal mammalian development.

Cytoskeletal derangements have also been reported, including altered blastomere shape and size, spindle abnormalities, and microtubule disassembly. These disruptions can interfere with major embryonic transitions and cell fate specification.^27^^,^^28^

In this study, we focused on the impact of vitrification on two cellular components whose interplay contributes to epigenetic remodeling and maintenance during embryo development: mitochondria and cytoplasmic lattices. Mitochondria serve as a primary interface between the environment and the epigenome, providing key metabolites necessary for epigenetic modifications during embryonic genome activation (EGA).^29^^,^^30^ Consequently, perturbations in mitochondrial structure and activity have the potential to significantly alter long-term embryo development by inducing heritable changes in the epigenome.^30^^,^^31^ Notably, mitochondrial structure and function have been shown to be disrupted in post-thawed human endometrial tissue^32^ and reproductive cells of various species^33^ following cryopreservation, which further motivated our study. Regarding cytoplasmic lattices, these filamentous structures store essential proteins involved in DNA methylation and demethylation during embryo development, ensuring proper epigenetic reprogramming.^34^ Although the impact of cryopreservation on cytoplasmic lattices remains unknown, studies have shown that disruption of lattices-associated proteins has been linked to severe impairments in EGA, causing developmental delays and imprinting defects, which are linked to imprinting disorders in humans.^34^^,^^35^

**Figure 1:**
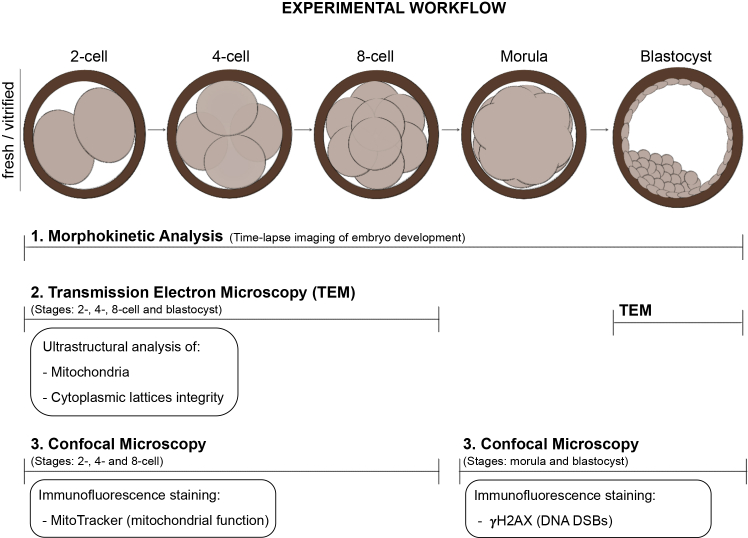
Experimental workflow of the study (1) Morphokinetic analysis of fresh and vitrified embryos to evaluate developmental progression from the 2-cell stage to the blastocyst stage. (2) Ultrastructural assessment of mitochondrial and cytoplasmic lattice integrity by transmission electron microscopy (TEM) performed at 2-, 4-, 8-cell, and blastocyst stages. (3) Evaluation of mitochondrial distribution and function at 2-, 4-, and 8-cell stages, and assessment of H2AX phosphorylation at morula and blastocyst stages, by immunofluorescence staining using a spinning-disk confocal microscope.

Using a mouse model and advanced imaging techniques, we conducted a comprehensive descriptive study to investigate the effects of a widely used vitrification protocol on early embryo development, with a particular focus on mitochondrial structure and function as well as the integrity of cytoplasmic lattices (see Figure 1 for overall workflow of our study). Our findings reveal that vitrification causes significant delays in early embryonic development, likely due to impaired mitochondrial function and disruption in cytoplasmic lattices abundance during the initial cell divisions.

## Results

### Time-lapse monitoring reveals notable delays in the developmental rhythm of vitrified mouse embryos

**Figure 2:**
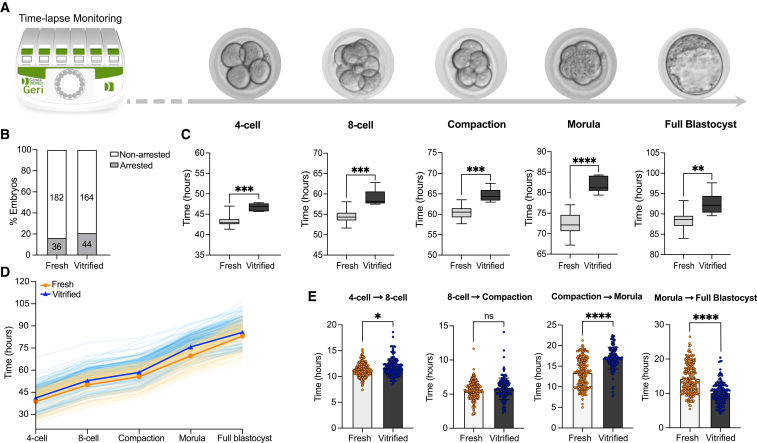
Time-lapse monitoring reveals notable delays in the developmental rhythm of vitrified mouse embryos (A) Developmental progression of fresh and vitrified embryos was monitored using a GERIⓇ time-lapse incubator. Embryos were vitrified and warmed at the 2-cell stage. Representative time-lapse images show the morphological appearance of mouse embryos at each developmental stage (left to right), captured at 10× magnification. (B) Percentage of non-arrested versus arrested embryos in fresh and vitrified groups. Embryonic arrest was defined as the absence of cellular division for ≥24 h. (C) Time (in hours) for fresh and vitrified embryos to reach specific stages: 4-cell, 8-cell, compaction, morula, and full blastocyst. Data shown as box and whisker plots, with medians, interquartile ranges (boxes), and minimum/maximum values (whiskers). (D) Developmental trajectories of individual embryos from 4-cell to full blastocyst. Each line represents a single embryo: yellow (fresh) and blue (vitrified), with group means in orange and dark blue, respectively. (E) Stage-specific durations (in hours) between developmental transitions. Each dot represents one embryo. Data shown as mean ± SD. Statistical comparisons in (C) and (E) were performed using an unpaired two-tailed Mann-Whitney test: (ns) > 0.05, ∗∗*p* < 0.01, ∗∗∗*p* < 0.001, ∗∗∗∗*p* < 0.0001. All images used the same cohort of embryos (218 fresh and 208 vitrified), derived from three independent experimental batches. Illustration of GERIⓇ incubator created in Affinity Designer. See also Figures S1 and S2.

To compare the developmental kinetics between fresh and vitrified embryos, we used a time-lapse imaging system to monitor progression from the 2-cell stage to the blastocyst stage (Figure 2A). Vitrification and warming were performed at the 2-cell stage, a critical point in mouse preimplantation development that coincides with the onset of EGA. This early stage also provides an opportunity to assess developmental progression and evaluate the impact of vitrification on early embryonic development within the limited time frame of *in vitro* culture.

The post-thaw survival rate of 2-cell stage mouse embryos was consistently high across experimental batches, ranging from 94.7% to 95.2% (Figure S1), comparable to reported Cryotop survival rates in human embryos.^36^^,^^37^^,^^38^

We first evaluated the rate of embryonic arrest in fresh versus vitrified embryos. Embryos that failed to progress for at least 24 h were considered arrested. Although a slight increase in embryonic arrest was observed in the vitrified group, this difference was not statistically significant when compared to the fresh group (Figure 2B).

Using a time-lapse Geri incubator, fresh and vitrified embryos were individually tracked, and the exact time required to reach each developmental stage was recorded. Significant developmental delays were observed in vitrified embryos compared to fresh embryos. On average, delays ranged between 3 and 4 h per developmental stage, with the most substantial cumulative delay (approximately 9 h) observed by the time embryos reached the morula stage (Figure 2C). To further illustrate this developmental delay, the progression of each individual embryo is shown in an alternative format in Figure 2D.

To investigate whether the observed delay in vitrified embryos was a global developmental shift or stage-specific, we performed an interval-specific analysis (Figure 2E). For each embryo, we calculated the duration of key stage transitions: 4-cell to 8-cell, 8-cell to compaction, compaction to morula, and morula to full blastocyst. Vitrified embryos required, on average, one additional hour to transition from the 4-cell to 8-cell stage compared to fresh embryos (11.15 h vs. 11.58 h). No significant difference was observed between groups for the 8-cell to compaction interval (5.58 h vs. 5.75 h). In contrast, vitrified embryos showed a substantial delay in the compaction to morula transition, taking nearly 4 h longer than fresh embryos (13.24 h vs. 17.00 h). Interestingly, this trend reversed in the morula to blastocyst transition, where vitrified embryos developed more rapidly, reaching the blastocyst stage approximately 3 h earlier than fresh counterparts (10.00 h vs. 13.60 h).

Despite these differences in developmental timing, blastocyst expansion was not visibly compromised by vitrification. As an indicator of developmental progression, the diameter of fresh and vitrified blastocysts was assessed, by measuring their maximum width (excluding zona pellucida).^39^^,^^40^ There was no significant difference in blastocyst diameter between groups, with average diameters of 104.7 μm (fresh) and 103.9 μm (vitrified) (Figure S2).

Taken together, although the overall developmental progression of vitrified embryos was delayed compared to fresh controls, interval-specific analysis revealed that the delay was not uniformly distributed across developmental stages. In particular, the accelerated transition from morula to blastocyst in vitrified embryos suggests that vitrification may alter the dynamics of specific transitions rather than simply shifting the entire developmental timeline.

### Vitrification alters morphometric parameters and may induce cell damage spots in 2-cell stage embryos

During the vitrification and warming process, embryos are exposed to fluctuations in osmotic pressure caused by the high concentrations of cryoprotectants used. In clinical settings, embryo quality and viability are typically assessed based on morphological characteristics including membrane integrity, blastomere survival, and cytoplasmic appearance. In this study, we performed a quantitative analysis to compare fresh and vitrified embryos by evaluating morphometric parameters such as blastomere surface area and volume.

**Figure 3:**
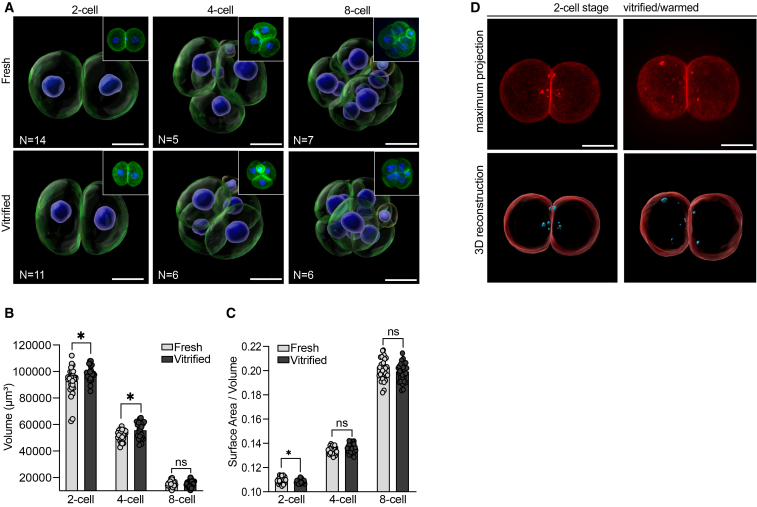
Vitrification alters morphometric parameters and may induce cell damage spots in 2-cell stage embryos (A) 3D reconstructions of fresh and vitrified embryos labeled with phalloidin (green) and DAPI (blue). Above each 3D reconstruction, a representative maximum projection of each embryo is shown (top right). (B) Graph representing blastomere volume (μm^3^). (C) Graph representing surface area-to-volume (S/V) ratio per blastomere. Each dot corresponds to one blastomere in a 2-cell, 4-cell, or 8-cell embryo. Data are represented as mean ± SD. Data were analyzed using an unpaired two-tailed Mann-Whitney test: (ns) > 0.05, ∗∗*p* < 0.01, ∗∗∗*p* < 0.001, ∗∗∗∗*p* < 0.0001. The number of embryos used is indicated in the figures. (D) Maximum projections and 3D reconstructions of fresh (not shown, *n* = 10) and vitrified (*n* = 8) 2-cell embryos labeled with Rhodamine-phalloidin (red). F-actin spots are highlighted in blue. Scale bars, 10 μm.

To achieve this, embryos were stained with phalloidin, an F-actin marker that reliably labels the cell membrane boundaries of blastomeres. Using Imaris software, the surfaces of individual blastomeres were rendered, and 3D reconstructions are shown in Figure 3A.

Like human embryos, mouse embryos exhibit an increase in the surface area-to-volume (S/V) ratio with each cell division, enhancing the efficiency of nutrient diffusion and waste removal. This developmental trend is observed in Figures 3B and 3C under fresh conditions. However, after thawing, 2-cell stage vitrified embryos exhibited a significant decrease in the S/V ratio compared to fresh embryos. This decrease likely reflects the effects of cryoprotectants, which are gradually diluted during warming to prevent osmotic shock. As the embryo rehydrates and begins to expand, its S/V ratio decreases. By the 4-cell and 8-cell stages, the S/V ratios were comparable between fresh and vitrified embryos.

Interestingly, as shown in Figure 3D, we also observed multiple spots of increased fluorescent signal from phalloidin. These F-actin-rich spots were found exclusively in vitrified 2-cell embryos, located within the cytosol near the cellular membrane. Accumulation of actin has been reported as part of a stress response at sites of cellular damage, to trigger activation and recruitment of cellular repair mechanisms.^41^^,^^42^^,^^43^ Based on these observations, we can only speculate that these actin-rich spots may represent a vitrification-induced stress response aimed at restoring the embryos’ cytoskeletal structure.

### Vitrification disrupts cytoplasmic lattices and mitochondrial structure during early mouse embryo development

For a detailed examination of the embryo’s cellular ultrastructure, transmission electron microscopy (TEM) was applied on both fresh and vitrified embryos, at the 2-cell, 4-cell, and 8-cell.

**Figure 4:**
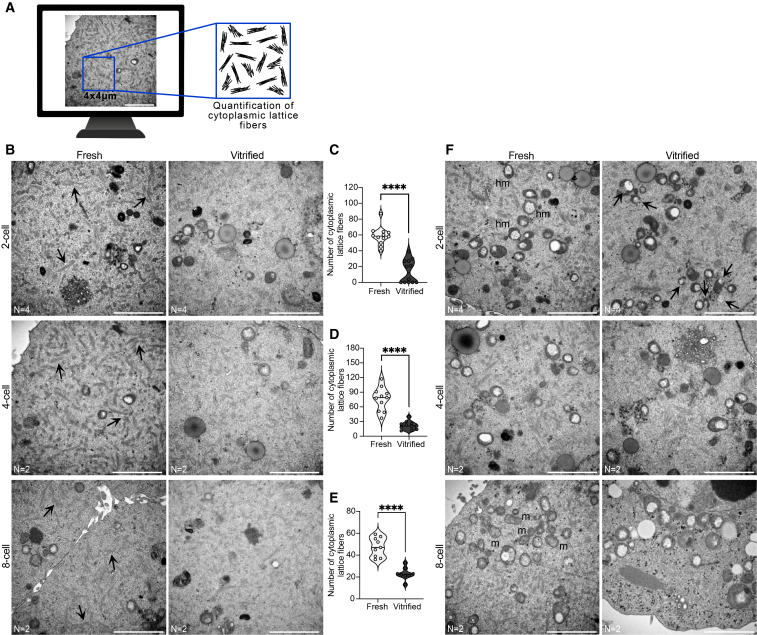
Vitrification disrupts cytoplasmic lattices and mitochondrial structure during early mouse embryo development (A) Schematic representation of cytoplasmic lattices quantification within a 4 × 4 μm region of interest (ROI). Depending on the developmental stage, micrographs from different regions of the embryo were analyzed: three micrographs for 2-cell stage embryos and six to eight micrographs for 4-cell and 8-cell stage embryos. (B) Representative transmission electron micrographs of fresh and vitrified 2-cell, 4-cell, and 8-cell embryos, illustrating the distribution and abundance of cytoplasmic lattice fibers. (C–E) Quantification of cytoplasmic lattice fibers in fresh versus vitrified embryos at each developmental stage. Each dot represents one analyzed micrograph. Results are presented as violin plots, where the width represents the distribution of values and the central line indicates the median. Statistical comparisons were performed using an unpaired two-tailed Mann-Whitney test: ns, *p* > 0.05; ∗*p* < 0.05; ∗∗*p* < 0.01; ∗∗∗*p* < 0.001; ∗∗∗∗*p* < 0.0001. (F) Transmission electron micrographs of fresh and vitrified 2-cell, 4-cell, and 8-cell embryos illustrating mitochondrial structure. Arrows indicate damaged mitochondria. hm, hooded mitochondria; m, maturing mitochondria. Scale bars, 2 μm. Number of embryos analyzed is indicated below the micrographs. See also Figure S3.

The first notable observation was the altered abundance and integrity of cytoplasmic lattices in vitrified embryos. To quantify cytoplasmic lattices density, a region of interest (ROI) was defined, and the same ROI was used to analyze all TEM images. As illustrated in Figure 4A, only cytoplasmic lattice fibers within the defined ROI were quantified.

At the 2-cell stage, the cytoplasm of fresh embryos was densely filled with cytoplasmic lattice fibers, while in vitrified embryos these fibers were significantly reduced or completely absent (Figures 4B and 3C). At the 4-cell stage, cytoplasmic lattices remained well-defined and visible in fresh embryos, while in vitrified embryos, they were significantly reduced and structurally altered, making individual filaments difficult to distinguish (Figures 4B and 4D). By the 8-cell stage, cytoplasmic lattices remained evident in fresh embryos, but in reduced amounts compared to earlier developmental stages. In vitrified embryos, these lattices were further reduced and exhibited a less distinct structure (Figures 4B and 4E).

During early embryo development, hooded mitochondria are the predominant type.^44^^,^^45^ Consistent with this, both fresh and vitrified embryos at the 2-cell stage displayed round-shaped mitochondria with a prominent vacuole-like space within the mitochondrial matrix, common characteristics of hooded mitochondria. Interestingly, in vitrified embryos, damaged mitochondria were observed with clear disruption of the mitochondrial outer membrane integrity (Figures 4F and S3). At the 4-cell stage, hooded mitochondria remained the predominant type, and no significant structural differences were observed between fresh and vitrified/warmed embryos (Figure 4F). By the 8-cell stage, fresh embryos displayed a higher number of mature mitochondria with a typical elongated structure. In contrast, most mitochondria from vitrified embryos appeared enlarged, with highly distended vacuole spaces that occupied most of the mitochondrial volume—a potential sign of slow mitochondrial maturation or degeneration (Figure 4F).

### Impact of vitrification on mitochondrial structure and cytoplasmic lattices in the blastocyst stage

Previous studies have reported poorer perinatal and obstetric outcomes following frozen blastocyst transfer,^6^^,^^46^ suggesting that later-stage embryos may also be vulnerable to adverse effects from cryopreservation. Therefore, we also performed TEM on both fresh and vitrified blastocysts (day 5 of development). The vitrified group was further divided into two subgroups: blastocysts vitrified at the 2-cell stage and those vitrified at the blastocyst stage. This allowed us to investigate whether the timing of vitrification influences blastocyst quality.

**Figure 5:**
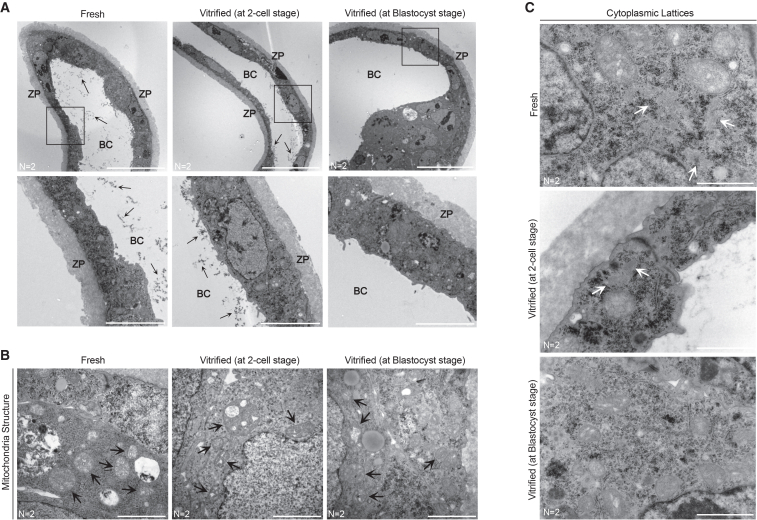
Impact of vitrification on mitochondrial structure and cytoplasmic lattices in the blastocyst stage (A−C) Representative transmission electron micrographs of fresh blastocysts, blastocysts vitrified at the 2-cell stage, and blastocysts vitrified at the blastocyst stage. (A) Depicts the presence (arrows) or absence of electron-dense materials within the blastocele, with insets providing magnified views of the outlined regions. (B) Illustrates mitochondrial structure, with arrows highlighting well-developed cristae in fresh embryos and poorly developed cristae in vitrified embryos. (C) Shows the presence (indicated by arrows) or absence of cytoplasmic lattice fibers. Scale bars: 20 μm (top) and 5 μm (bottom) in (A) and 2 μm in (B and C). Number of embryos analyzed is indicated below each micrograph. ZP, zona pellucida; BC, blastocele.

Our first notable observation was the presence of electron-dense material within the blastocele (Figure 5A). Initially, we suspected these could be artifacts from the preparation or sectioning process. However, since these materials were exclusively found within the blastocele, we ruled out this possibility. While the exact nature of these electron-dense materials remains unclear, previous literature suggests they may be protein aggregates secreted by trophoblast cells to support blastocyst development.^47^^,^^48^^,^^49^^,^^50^ Interestingly, these materials were observed in fresh blastocysts and those vitrified at the 2-cell stage but were absent in blastocysts vitrified at the blastocyst stage.

Mitochondrial damage was observed in both the vitrified subgroups (Figure 5B). Mitochondria in vitrified blastocysts exhibited poorly developed cristae and signs of vacuolization, indicative of compromised function and swelling. In contrast, fresh blastocysts displayed well-developed cristae, reflecting good mitochondrial integrity.

Cytoplasmic lattices were observed in fresh blastocysts, with well-organized and clearly defined filaments (Figure 5C). In blastocysts vitrified at the 2-cell stage, cytoplasmic lattices were also present but in smaller agglomerates and at a lower abundance. In blastocysts vitrified at the blastocyst stage, cytoplasmic lattices were not apparent.

These findings highlight the impact of vitrification on mitochondrial and cytoplasmic lattices integrity, even at a later stage of development. Moreover, our results suggest that the timing of vitrification, whether at the 2-cell stage, or at the blastocyst stage, can have differential effects on blastocyst quality. Therefore, the timing of vitrification should be carefully considered to optimize the preservation of blastocyst integrity and minimize cryopreservation-induced damage.

### Vitrification disrupts mitochondrial volume and membrane potential during early mouse embryo development

From ovulation until the morula stage in mammals, oocyte and early embryo development rely heavily on the mitochondria to meet their metabolic needs.^51^ Variations in mitochondrial content and distribution have been associated with changes in embryo developmental rates in previous studies.^52^ These variations are linked with adaptive mechanisms to help maintain a spatial balance of ATP supply in response to fluctuating energy demands during embryonic progression.^53^

Given the developmental delay observed in vitrified embryos compared to fresh, we aimed to assess how mitochondrial distribution and function are affected following vitrification/warming. To evaluate this, fresh and vitrified embryos at the 2-cell, 4-cell, and 8-cell stages were stained with MitoTracker Red, a potential-dependent dye that accumulates in mitochondria based on their membrane potential.

**Figure 6:**
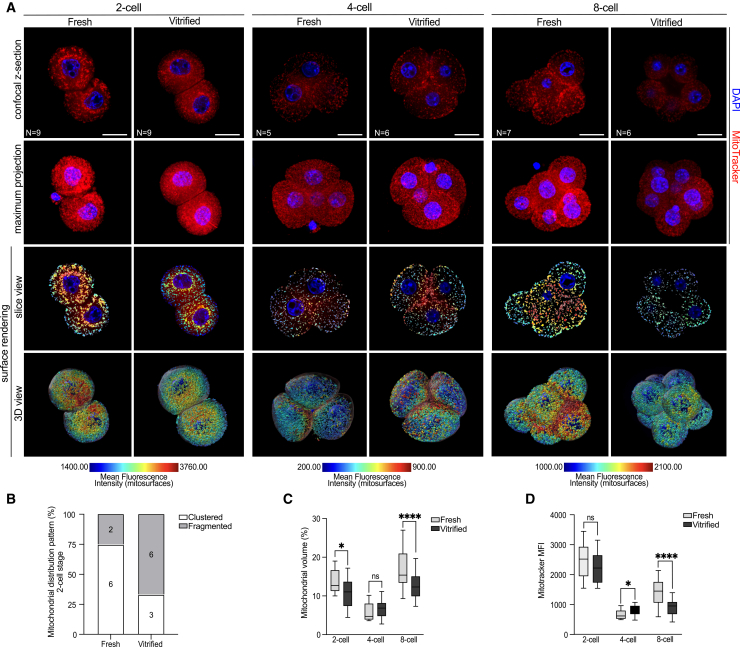
Vitrification disrupts mitochondrial volume and membrane potential during early mouse embryo development (A) Representative confocal z sections (middle, z-slice) and maximum projections (top two rows) of fresh and vitrified 2-cell, 4-cell, and 8-cell stage embryos, stained for mitochondria (MitoTracker, red) and DNA (DAPI, blue). Slice view mode and 3D reconstruction (third and fourth rows) show rendered mitochondrial surfaces from 60 to 90 optical sections. MitoTracker intensity is represented as a heatmap with a numerical color-coded scale shown below. Scale bars, 10 μm. (B) Mitochondrial distribution in fresh and vitrified 2-cell stage embryos was classified as either clustered or fragmented. Numbers indicate the embryos classified under each category. Patterns in 4-cell and 8-cell embryos were identical for both fresh and vitrified conditions and thus were not included in the analysis. (C and D) Graphs showing mitochondrial volume (per blastomere) and MitoTracker fluorescence intensity (MFI, per blastomere) in fresh and vitrified embryos, respectively. Results are expressed as box and whisker plots, displaying medians and interquartile ranges (boxes), and minimum and maximum values (whiskers). Data were analyzed using an unpaired two-tailed Mann-Whitney test: (ns) > 0.05, ∗∗*p* < 0.01, ∗∗∗*p* < 0.001, ∗∗∗∗*p* < 0.0001. The number of embryos analyzed is indicated in the first row.

In fresh 2-cell embryos, mitochondria were predominantly distributed in the perinuclear area and cytoplasm in a clustered pattern (Figures 6A and 5B). In contrast, most vitrified embryos exhibited a more dispersed mitochondrial distribution within the cytoplasm (Figures 6A and 5B). The functional significance of mitochondrial clustering in early embryo development remains debated. Some studies suggest that clustering reflects an inability of embryos to develop *in vitro*,^54^ while others link it to enhanced ATP production for rapid energy supply.^44^ At later stages (4-cell and 8-cell), mitochondrial distribution appeared similar in both fresh and vitrified embryos, with mitochondria evenly dispersed throughout the cytoplasm of each blastomere (Figure 6A).

To further investigate mitochondrial changes, Imaris software was used to generate surface renderings of mitochondria. This analysis identifies each individual mitochondrion or mitochondrial network as a separate rendered surface. The average mitochondrial volume per blastomere was calculated and is presented in Figure 6A, in both slice view and 3D view modes for fresh and vitrified embryos. At the 2-cell stage, vitrified embryos showed a significant decrease in average mitochondrial volume compared to fresh embryos. At the 4-cell stage, mitochondrial volume increased in vitrified embryos, though this change was not significant. Interestingly, by the 8-cell stage, vitrified embryos showed a pronounced decrease in mitochondrial volume compared to fresh embryos (Figure 6C).

In addition to volume, mitochondrial fluorescence intensity was measured for each rendered mitochondrial surface, referred to as “mitosurfaces.” Heatmaps representing MitoTracker intensity are shown in Figure 6A. At the 2-cell stage, MitoTracker intensity was comparable between fresh and vitrified embryos. However, at the 4-cell stage, MitoTracker intensity significantly increased in vitrified embryos compared to fresh, possibly suggesting a metabolic compensation by the embryos following vitrification. By 8-cell stage, MitoTracker intensity markedly decreased in vitrified embryos, consistent with the observed reduction in mitochondrial volume (Figures 6C and 6D).

Taken together, these findings demonstrate the potential consequences of vitrification on mitochondrial organization and membrane potential during early embryonic development.

### Vitrification increases γH2AX signal in late preimplantation embryos

**Figure 7:**
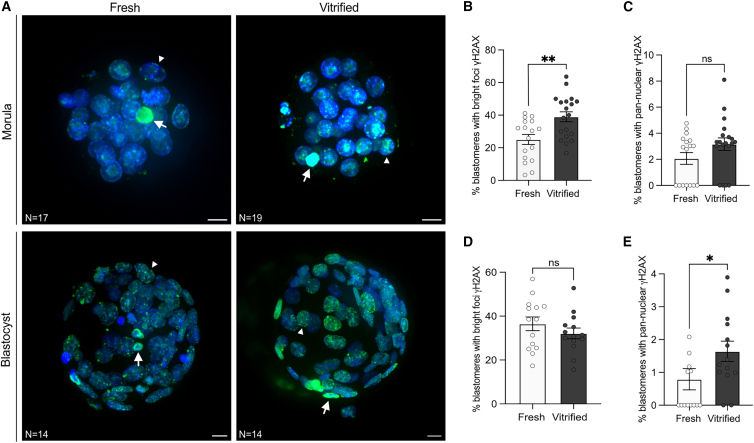
Vitrification increases γH2AX signal in late preimplantation embryos (A) Representative maximum projections of fresh and vitrified morulae and blastocysts stained for γH2AX (green, DNA double-strand break marker) and DNA (DAPI, blue). Arrowheads indicate blastomeres showing bright γH2AX foci, and arrows indicate nuclei with pan-nuclear signal. (B and D) Quantification of the percentage of blastomeres with bright γH2AX foci in fresh versus vitrified embryos. (C and E) Quantification of the percentage of blastomeres with pan-nuclear γH2AX labeling in fresh versus vitrified embryos. Each dot represents one embryo. Data are presented as mean ± SD. Statistical comparisons were performed using an unpaired two-tailed Mann-Whitney test: ns > 0.05, ∗∗*p* < 0.01, ∗∗∗*p* < 0.001, ∗∗∗∗*p* < 0.0001. The number of embryos analyzed is indicated in each image. Scale bars, 10 μm.

To determine whether vitrification at the 2-cell stage affects nuclear stress in developing mouse embryos, we performed γH2AX immunofluorescence at morula and blastocyst stages. These stages are characterized by high proliferative activity, extensive chromatin remodeling, and crucial epigenetic changes associated with EGA, making them particularly sensitive to oxidative or genotoxic stress.^55^ Two distinct nuclear γH2AX patterns were observed: bright foci, which typically indicate the presence of DNA double-strand breaks, and pan-nuclear signal, often associated with widespread H2AX phosphorylation under conditions of replication or apoptotic stress (Figure 7A).

Quantification was performed by normalizing the number of γH2AX-positive nuclei to the total number of 4', 6-diamidino-2-phenylindole (DAP)-stained nuclei per embryo (Figure 7B). A summary of the absolute counts for each parameter (cell number, bright foci, and pan-nuclear signal per embryo) is provided in Figure S5.

Vitrified morula showed a significant increase in the percentage of nuclei displaying bright γH2AX foci compared with fresh controls, while the frequency of nuclei with pan-nuclear labeling also tended to be higher but was not statistically significant. By the blastocyst stage, the frequency of bright foci was similar between groups, but vitrified embryos displayed a modest yet significant rise in nuclei with pan-nuclear γH2AX signal.

These results indicate that vitrification modifies the pattern of γH2AX activation in later preimplantation stages. The shift from localized foci at the morula stage to broader γH2AX labeling at the blastocyst stage suggests a transition from focal DNA damage responses toward more global chromatin phosphorylation, potentially reflecting altered DNA repair dynamics or sustained nuclear stress.

## Discussion

In this study, we used a mouse model to investigate the effects of vitrification on preimplantation embryo development and subcellular structures. This work provides a detailed descriptive characterization of how cryopreservation influences developmental dynamics and subcellular organization, serving as a foundation for future functional studies. While our findings provide insights relevant to reproductive medicine, direct extrapolation to human embryos should be approached cautiously. Traditionally, cryopreservation techniques have been evaluated based on observable morphological changes and clinical outcomes, such as *in vitro* embryo survival, implantation, and birth rates. However, our findings highlight that such assessments may overlook subtle yet significant disruptions to early developmental processes.

To the best of our knowledge, this is the first study to provide a comprehensive comparison of developmental kinetics between vitrified and fresh mouse embryos, revealing significant differences across all preimplantation stages. While vitrified embryos exhibited an overall developmental delay, stage-specific analyses showed that these delays were not uniformly distributed. This non-uniform pattern suggests that vitrification may not merely slow down overall development but rather alter the timing and dynamics of specific morphogenetic events. Previous work in bovine embryos has shown that early cleavage timing influences embryo synchrony and is tightly linked to transcriptomic profiles at the blastocyst stage,^56^ emphasizing the potential developmental consequences of disrupted morphokinetics in vitrified embryos.

One possible explanation for the accelerated morula to blastocyst transition in vitrified mouse embryos is the activation of compensatory mechanisms. According to previous literature, mammalian embryos possess a degree of developmental plasticity that enables self-correction through gene expression adjustments, cytoskeleton reorganization, and flexible blastomere behavior.^57^^,^^58^ These adaptive processes may allow vitrified embryos to regain developmental synchrony by the morula stage. Alternatively, the acceleration may reflect vitrification-induced changes in cell adhesion and compaction dynamics. Proper morula formation depends on adhesion molecules such as E-cadherin and tight junction integrity,^59^^,^^60^^,^^61^^,^^62^ both of which have been reported as disrupted in vitrified embryos of other species.^63^^,^^64^ If compaction is incomplete or unstable, it could reduce the mechanical resistance needed for cavitation, leading to earlier blastocele formation. In this scenario, the observed acceleration in blastocyst formation may not reflect true developmental progress but rather a shift in the timing of cavity formation due to altered cell behavior. Further investigation into adhesion molecules and junctional integrity is needed to confirm this possibility.

In this study, mouse embryos were vitrified using an open system commonly employed in fertility clinics, wherein embryos, embedded in cryoprotectant solution, come into direct contact with liquid nitrogen. Interestingly, distinct F-actin spots were observed near the cellular membrane exclusively in vitrified 2-cell embryos. F-actin accumulation has been previously reported to signal cellular damage, aiding repair at wound sites and in response to DNA damage.^43^^,^^65^ These F-actin spots might result from mechanical friction between the embryos and the vitrification device, or from osmotic stress caused by freezing and warming. Osmotic stress and acute changes in cell volume are well-documented to induce cytoskeleton rearrangements and increase cellular F-actin in various cell types.^66^^,^^67^^,^^68^^,^^69^^,^^70^ Notably, these F-actin spots were absent in vitrified embryos at later developmental stages (4-cell and 8-cell stages), suggesting that the observed actin accumulation at the 2-cell stage may reflect a transient cellular repair response to vitrification-induced damage, which is subsequently resolved as development progresses.

Vitrification was performed at the 2-cell stage, coinciding with the major wave of EGA in mice.^71^ EGA is a critical event in mammalian reproduction, during which the embryo assumes control of gene expression to regulate cell differentiation and further development. Failure to remodel the epigenome or activate embryonic transcription can result in implantation failure and developmental consequences, either *in utero* or later in life.^71^^,^^72^ Successful reprogramming and embryo development rely on energy and metabolic cofactors produced by mitochondrial metabolism. As dynamic biosensors, mitochondria are highly sensitive to environmental perturbations, and their dysfunction have been shown to impair embryonic, fetal, and placental development.^73^^,^^74^

Early stage embryos are enriched in “hooded” mitochondria, which are often described as “underdeveloped” or a “transition type mitochondria,” as they represent an intermediate stage in mitochondrial maturation.^44^^,^^45^^,^^75^^,^^76^^,^^77^ These mitochondria provide initial glycolytic metabolic support, which transitions to oxidative phosphorylation as the embryo’s metabolic demands increase.^44^^,^^75^^,^^76^^,^^77^ Indeed, hooded mitochondria were prevalent in 2-cell stage embryos, but vitrified embryos showed clear signs of mitochondrial membrane rupture. This might reflect rapid temperature fluctuations and osmotic stress during vitrification/warming, which can destabilize membrane lipid structure and composition—a common cause of cellular cryodamage.^78^ Mitochondrial membrane rupture may allow leakage of critical mitochondrial components, impairing energy production. This aligns with the observed decrease in mitochondrial membrane potential at the 8-cell stage (Figures 6A and 6D). Interestingly, vitrified 8-cell embryos also exhibited a higher number of hooded mitochondria compared to fresh embryos, potentially reflecting delayed mitochondrial maturation, or a broader developmental delay. Mitochondrial structure was also compromised at the blastocyst stage, regardless of whether vitrification occurred at the 2-cell or blastocyst stage. We observed mitochondrial swelling and poorly developed cristae, features similarly reported in vitrified blastocysts from sheep, porcine, and bovine.^79^^,^^80^^,^^81^

Following fertilization, the early embryo relies almost exclusively on maternally inherited mitochondria, as *de novo* mitochondrial DNA replication does not occur until the blastocyst stage.^82^^,^^83^^,^^84^^,^^85^ During cleavage divisions, mitochondrial content is partitioned into daughter cells without mtDNA replication.^82^^,^^83^^,^^84^^,^^86^^,^^87^ Because mitochondrial biogenesis is minimal during these stages, any loss in mitochondrial content or quality cannot be readily compensated, supporting the existence of a functional “mitochondrial setpoint” essential for successful preimplantation development. Our findings indicate that vitrification at the 2-cell stage leads to an early mitochondrial insult, characterized by reduced mitochondrial volume, which could reflect activation of mitophagy shortly after warming. By the 4-cell stage, we observed a significant rise in MitoTracker intensity in vitrified embryos. Although mitochondria biogenesis is limited during early development, embryos retain the ability to adjust the functional activity of their existing mitochondria. This includes metabolic reprogramming, upregulation of nuclear-encoded mitochondrial genes, and alterations in mitochondrial dynamics.^30^^,^^53^^,^^88^^,^^89^ The observed rise in MitoTracker intensity at the 4-cell stage may represent a compensatory metabolic response, wherein the embryo attempts to enhance mitochondrial function and energy production to support developmental progression despite earlier damage. However, the short duration of the 4-cell stage may limit the extent of this compensation. By the 8-cell stage, both mitochondrial volume and MitoTracker intensity were markedly reduced in vitrified embryos, indicating a progressive decline in mitochondrial capacity. Further research is needed to clarify whether the observed changes in mitochondria reflect a metabolic adaptation or a cellular stress response to cryopreservation.

A remarkable finding of this study relates to cytoplasmic lattices. These highly ordered arrays of fibers, first identified in the 1960s,^90^^,^^91^ have long remained enigmatic. Recent research by Jentoft et al. demonstrated that mammalian oocytes store proteins essential for early embryonic development on cytoplasmic lattices.^35^ Loss of these structures prevents the accumulation of these proteins and impairs early development, resulting in embryonic arrest, molar pregnancies, or offspring with imprinting disorders.^35^

Our data revealed that cytoplasmic lattices in vitrified embryos were significantly reduced in abundance and exhibited compromised structural integrity during the initial cleavage stages. At the blastocyst stage, cytoplasmic lattices were still observed in fresh embryos, although in much lower abundance compared to earlier cleavage stages. While cytoplasmic lattices in blastocysts remain poorly studied, they are known to be dynamic structures that undergo extensive changes in spatial organization coinciding with major developmental transitions such as fertilization, blastomere compaction, and blastocyst formation.^92^^,^^93^^,^^94^ Over time, as the embryo develops, these structures eventually fragment or disassemble.^92^^,^^93^^,^^94^ This is consistent with the observed reduction of cytoplasmic lattices abundance in fresh blastocysts. This reduction likely occurs because most maternal RNA and proteins have been degraded or used, and the zygotic genome now drives development.

In vitrified conditions, cytoplasmic lattices abundance was even lower or completely absent. Given that these structures were already observed in reduced quantities under fresh conditions, vitrification may have further degraded them. Whether this has a significant impact on blastocyst development and quality requires further investigation.

Previous studies in mouse oocytes and zygotes have shown that disruption of specific lattices-associated proteins can alter mitochondria morphology and distribution, as well as compromise energy production.^95^^,^^96^^,^^97^^,^^98^ However, the exact mechanisms by which cytoplasmic lattices contribute to mitochondrial function remains unclear. Further research is needed to determine whether disruption of cytoplasmic lattices plays a role in the observed mitochondrial dysfunction in vitrified embryos, and consequently, their developmental delays.

Lastly, consistent with previous reports in oocytes and embryos, vitrification also induced nuclear stress, as indicated by increased γH2AX signal at the morula and blastocyst stages. Similar DNA damage responses have been described following vitrification in mouse and porcine models, often associated with elevated reactive oxygen species (ROS) levels and reduced developmental competence.^99^^,^^100^^,^^101^^,^^102^ The shift from discrete γH2AX foci to pan-nuclear labeling observed here suggests that vitrification may trigger both localized DNA repair and broader chromatin phosphorylation events. This nuclear stress may arise from mitochondrial dysfunction and the concurrent loss of cytoplasmic lattices, which together may disrupt the metabolic and structural support required to maintain genomic stability during preimplantation development.

In conclusion, mitochondrial dysfunction, loss of cytoplasmic lattices, and evidence of nuclear stress together may underlie the disrupted developmental kinetics observed in vitrified mouse embryos. While these observations provide mechanistic insight into early embryonic responses to vitrification, their direct relevance to human embryos remains to be determined. Our results highlight the importance of continued mechanistic investigation into how cryopreservation impacts subcellular organization and early embryonic development, which could guide future improvements in assisted reproductive technologies.

### Limitations of the study

It is worth noting that, although our work provides intriguing insights into the impact of cryopreservation on embryo development, several issues remain to be addressed.

First, our research was conducted using a mouse experimental model, which may not fully reflect human embryonic development due to considerable species-specific differences in developmental timing, metabolism, and events such as EGA. While mice provide a highly controlled platform for mechanistic studies, additional research is needed to determine whether similar alterations occur in other mammalian models and to clarify their potential relevance for human embryos. In addition, we used a vitrification method (Kitazato Cryotop) developed and standardized for human clinical applications. While this system has been successfully adapted in mice and yields high post-warming survival and developmental rates, subtle differences in optimal cryoprotectant exposure times, cooling rates, or loading geometries between species cannot be excluded. These parameters may influence the magnitude of the observed subcellular effects. Future work comparing alternative vitrification workflows within the mouse model would be valuable to confirm the generality and reproducibility of our findings.

Second, part of our mitochondrial studies was performed on fixed embryos using confocal microscopy. While informative, this approach limits the ability to assess real-time mitochondrial behavior, as these organelles are highly responsive to the cell cycle, metabolic state, and environmental conditions. Although embryos in this study were tightly time-controlled, future research should incorporate live imaging techniques to more accurately capture mitochondrial dynamics during development.

Third, due to the technical complexity and time-intensive nature of TEM, our sample size for ultrastructural analysis was limited. In addition, although TEM provided valuable ultrastructural details, its two-dimensional nature may result in the loss of important information along the embryo’s depth. Further structural studies are also needed to explore the three-dimensional organization of cytoplasmic lattices following cryopreservation.

Finally, while this work focused on descriptive and ultrastructural analyses, functional validation remains an important next step. Molecular assays, including single-cell transcriptomics, genome-wide DNA methylation profiling, and histone modification analysis, could reveal how cryopreservation-induced structural changes impact gene expression and epigenetic programming during early development. Moreover, extending these analyses beyond the blastocyst stage will be critical to determine whether the early alterations we describe persist, are compensated during subsequent development, or lead to lasting consequences. Future work integrating embryo transfer experiments with post-implantation staging, as well as fetal and placental assessments, will help clarify whether the subcellular disruptions reported here translate into long-term developmental outcomes.

## Resource availability

### Lead contact

Requests for further information and resources should be directed to and will be fulfilled by the lead contact, Arturo Reyes Palomares (arturolrp@uma.es).

### Materials availability

This study did not generate new unique reagents.

### Data and code availability

• This paper does not report original code. All data associated with this study are present in the paper or supplemental information.
• Any additional information required do reanalyze the data reported in this paper is available from the lead contact upon request.

## Acknowledgments

We would like to express our sincere gratitude to the staff at the Animal Facility, Live-Cell Imaging Facility, Biomedicum Imaging Core Facility, and Electron Microscopy Core Facility of Karolinska Institutet for their invaluable technical assistance. In particular, we extend our thanks to Muntaha Fartoo for her assistance with animal experiments and embryo collection; Gabriela Imreh for her support with the embryo immunofluorescence protocol and confocal microscopy; Göran Månsson for his expertise and advice on confocal image analysis, particularly with Imaris software; and Dr. Lars Haag and Lisa Sjöwall for their help with embryo processing for transmission electron microcopy and image collection. We also wish to acknowledge Birgitta Lindqvist for her support in managing laboratory resources, handling orders, and ensuring proper management of the laboratory environment. We further acknowledge Merck AB Sweden for providing access to the Geri incubator for time-lapse imaging used in the study. The research leading to these results was funded by KI Research Foundation grants 2024-2025(2024-02566) and the 10.13039/501100004359Swedish Research Council Grants Open call 2023 (Medicine and Health) 2023-01872. K.A.R.-W. is supported by the 10.13039/501100002794Swedish Cancer Society, Radiumhemmets Research Funds and ALF grants from Region Stockholm. A.R.P. is supported by fundings of the Beatriz Galindo Program (BG23-00015) and PID2024-160756OA-I00 funded by the Spanish Ministry of Science, Innovation and Universities (MICIU/AEI, 10.13039/501100011033) by European Regional Development Fund (ERDF, “A way of making Europe”), the European Union.

## Author contributions

Conceptualization, M.T.B., K.A.R.-W., and A.R.P.; methodology, M.T.B. and A.R.P.; investigation, M.T.B., K.D., J.A.R.M., and V.S.; morphokinetics analysis, M.T.B. and K.D.; TEM analysis, M.T.B., J.A.R.M., V.S., and J.Z.; immunofluorescence analysis, M.T.B., J.A.R.M., and V.S.; visualization and writing – original draft, M.T.B; writing – review and editing, M.T.B., K.A.R.-W., and A.R.P.; funding acquisition, K.A.R.-W. and A.R.P.; supervision, K.A.R.-W. and A.R.P. All authors have read and approved the submission of the manuscript.

## Declaration of interests

The authors declare no competing interests.

## STAR★Methods

### Key resources table

**Table:** 

REAGENT or RESOURCE	SOURCE	IDENTIFIER
**Antibodies**		

Anti-phospho-Histone H2A.X (Ser139)	Sigma-Aldrich	Cat# 05-636; RRID:AB_309864
Alexa Fluor 488 Donkey Anti-Mouse IgG (H + L) Antibody	Thermo Fisher Scientific	Cat#A-21202; RRID:AB_141607

**Biological samples**		

Zygotes	Mice from this paper	N/A

**Chemicals, peptides, and recombinant proteins**		

Pregnant mare serum gonadotropin	MSD Animal Health	N/A
Human chorionic gonadotropin	MSD Animal Health	N/A
Vitrification and Warming Media	Kitazato	Cat# VT601 and VT602
MitoTracker™ Red CMXRos	Thermo Fisher Scientific	Cat# M7512
Alexa Fluor™ 488 Phalloidin	Thermo Fisher Scientific	Cat# A12379
Rhodamine Phalloidin	Thermo Fisher Scientific	Cat# R415
VECTASHIELD® Antifade Mounting Medium with DAPI	Vector Laboratories	Cat# H-1200-10
Paraformaldehyde 4%	Santa Cruz	Cat# sc-281692
EmbryoMax Advanced KSOM Medium	Sigma-Aldrich	Cat# MR-101-D
OVOIL	Vitrolife	Cat# 10029

**Experimental models: Organisms/strains**		

Mouse: C57BL/6J	PKL Facility, Karolinska Institutet	47
Mouse: CBA/J	PKL Facility, Karolinska Institutet	47

**Software and algorithms**		

Geri® Connect & Assess	Genea Biomedx	https://www.geneabiomedx.com/
Nikon NIS-Elements	Nikon Instruments	Version 4.20
Imaris	Oxford Instruments	Version 10.2
Image J	National Institutes of Health	Version 2.14.0
GraphPad Prism	GraphPad Software, Boston, Massachusetts USA	Version 10.0.0
Affinity Designer	Affinity	Version 2.0

**Other**		

Cryotop	Kitazato	Cat# 90613 and 90614

### Experimental model and study participant details

#### Husbandry and housing conditions of experimental animals

For this study, C57BL/6J (B6) female and CBA/J (CBA) male mice were housed in cages with free access to sterilized water and pelleted food in a temperature-controlled room, maintained under an artificial 12-hour light/12-hour dark cycle.

All animal experiments conducted in this study were approved by the Stockholm Ethical Committee for Animal Research (19705-2022) and complied with the Directive 2010/63/EU of the European Parliament on the protection of animals used for scientific purposes. Animal care and experimental procedures were performed and monitored at the Preclinical Laboratory 4 (PKL4) at Karolinska University Hospital, Huddinge, in accordance with accepted standards for humane animal care.

### Method details

#### Superovulation and mating

Superovulation was induced in 6- to 8-week-old female mice using a combination of pregnant mare serum gonadotropin (PMSG) and human chorionic gonadotropin (hCG). On Day 0, mice received an intraperitoneal injection of PMSG. Forty-eight hours later (Day 2), they were given hCG. Immediately after hCG injection, female mice were mated in a 1:1 ratio with proven fertile male mice. Successful mating was confirmed the following day (Day 3) by checking for the presence of a vaginal plug. Female mice were euthanized via cervical dislocation. See Figure S5 for a schematic overview.

#### Embryo collection and culture

From euthanized female mice, ovaries and associated oviducts were dissected. Zygotes were released from the ampullae into pre-warmed M2 medium and transferred to a hyaluronidase solution (HYASE^TM^-10X, Vitrolife) to facilitate removal of the surrounding cumulus cells. Zygotes were then washed in M2 medium, and cultured in pre-equilibrated EmbryoMax Advanced KSOM Medium (Sigma-Aldrich) covered with mineral oil (OVOIL^TM^; Vitrolife), and incubated at 37°C with 5% O2 and 6% CO2. See Figure S5 for a schematic overview. At this point, embryos were randomly assigned either to vitrification or to continued culture as fresh controls. Vitrified embryos were subsequently warmed and cultured under identical conditions as the fresh group. For each experimental batch, embryos were obtained from 10 superovulated females, and both experimental groups included embryos derived from each donor to control for inter-female variability. Sample sizes were determined based on the number of embryos obtained per collection round.

#### Embryo vitrification and warming

In the vitrified group, 2-cell mouse embryos were cryopreserved using the Cryotop Method developed by Kitazato.^11^^,^^12^ Briefly, embryos were incubated in Equilibration Solution (ES) for 10-15 minutes. ES contains a lower concentration of permeable cryoprotectants, which helps the embryos lose water gradually while beginning to take up the cryoprotectants. After an initial shrinkage, embryos returned to their original size and were transferred into Vitrification Solution 1 (VS1) for 30 seconds, followed by Vitrification Solution 2 (VS2) for another 30 seconds. VS1 and VS2 contain higher concentrations of cryoprotectants and often include additional components, such as sucrose, which creates an osmotic gradient to promote further dehydration. These steps ensure complete dehydration and adequate cryoprotectant permeation before rapid cooling. The embryos were then loaded onto the surface of the Cryotop strip with minimal volume and then plunged directly into liquid nitrogen.

For warming, the Cryotop strip was immersed in Thawing Solution 1 at 37°C for 1 minute until all embryos were released. Embryos were then transferred through a series of solutions at room temperature: Diluent Solution for 3 minutes, Warming Solution 1 for 5 minutes, and Warming Solution 2 for 1 minute. These steps ensure controlled rehydration of the embryos and removal of cryoprotectants. Upon completion of the warming procedure, embryos were transferred to pre-equilibrated EmbryoMax Advanced KSOM Medium, overlaid with mineral oil, for further culture.

Vitrified embryos were cultured for at least 2 hours to allow recovery before being subjected to additional experimental protocols. See Figure S5 for a schematic overview.

#### Time-lapse embryo culture

Fresh and vitrified 2-cell embryos were individually cultured in 16-microwell Geri® dishes, allowing the tracking of each embryo. They were then placed in a Geri® incubator for 110-120 hours post-insemination. Brightfield images were captured every 5 minutes at a resolution of 2 pixels/μm, from the 2-cell stage until the blastocyst stage. Embryo development was analyzed using Geri® Connect and Assess 1.0 software. Time intervals (in hours) from the 2-cell stage to the blastocyst stage were annotated and compared between fresh and vitrified embryos.

To normalize the morphokinetic data, an initial normalization step was performed by defining the time of hCG injection as the starting point for both fresh and vitrified groups. This provided a consistent reference for all embryos, minimizing variability due to differences in fertilization timing, which is difficult to determine precisely *in vitro*. An additional normalization step was applied to account for the delay caused by cryopreservation. Specifically, the time embryos remained frozen (approximately 2 hours) was subtracted from the total time each vitrified embryo took to reach each developmental stage. This adjustment ensured that only the time spent in culture post-thawing was considered in the analysis.

#### Immunofluorescence staining

Embryos were incubated at 37°C for 1 hour in 400 nM MitoTracker Red CMXRos (Invitrogen) diluted in pre-equilibrated KSOM. After incubation, embryos were washed in KSOM for 1 hour at 37°C and then fixed in 4% paraformaldehyde (Sigma-Aldrich) for 20 min at room temperature (RT). Embryos were then washed 3 × 5 minutes in PBA (PBS with 0.5% BSA, Sigma-Aldrich) at RT. Next, embryos were incubated in blocking solution (10% FBS in PBS, Sigma Aldrich) for 2 hours at RT or overnight at 4°C. Following blocking, embryos were incubated with Alexa Fluor^TM^ 488 Phalloidin (Invitrogen) diluted 1:200 in blocking solution while protected from light, followed by 3 × 10 min washes in PBA. For γH2AX staining, embryos were incubated overnight at 4°C with Anti-phospho-Histone H2A.X (Ser139) (Sigma-Aldrich) diluted 1:200 in blocking solution, washed (3×5 min in PBA), and incubated with Alexa Fluor^TM^ 488 Donkey Anti-Mouse IgG (H+L) (Invitrogen, 1:200) for 1 h at RT, protected from light, followed by 3×10 min washes in PBA. Embryos were mounted onto μ-Slide 8-Well Glass Bottom (Ibidi) in droplets of VECTASHIELD Antifade Mounting Medium with DAPI (Vector Laboratories).

#### Confocal microscopy

Embryos were imaged using a Nikon Ti2 inverted spinning disk confocal microscope with 60× water objective, controlled by NIS-Elements software at the Live Cell Image Core Facility (LCI) of Karolinska Institutet. To ensure consistency and reproducibility, all embryos were imaged using the same microscope settings, including laser intensity and exposure time. Images were obtained using 405-, 477- and 546-nm lasers. Z-stack images were acquired sequentially for each channel at 1 μm intervals along the embryo’s depth.

#### Mitochondrial function analysis

The acquired Z-stacks were opened in their native format in Imaris software (version 10.1, Oxford Instruments), where 3D reconstructions were automatically generated. Deconvolution of the MitoTracker channel was performed using a Gaussian filter to reduce noise. Subsequently, the images were converted to surface renderings using the “Surfaces” model for mitochondria and the “Cell” model for embryo blastomeres.

Imaris identified each individual mitochondrion as a surface by detecting MitoTracker fluorescence intensity through local contrast. Surface rendering parameters were manually adjusted for each embryo to ensure accurate measurements, avoiding the use of a fixed threshold.

Embryo blastomeres were rendered using the automatic Cell recognition function, based on fluorescence intensity of the cell boundary stained with phalloidin. In cases where automatic segmentation proved inadequate, manual rendering was performed by profiling the phalloidin signal every third Z-stack section along the embryo’s length, using a vertex spacing of 2.76 μm.

Once surface renderings were complete, parameters such as mitochondrial volume and intensity, as well as blastomere volume and area, were extracted for quantification. Mitochondrial volume was defined as the proportion of a blastomere’s volume occupied by mitochondria and was calculated by dividing the total mitochondrial volume by the volume of the corresponding blastomere (μm^3^).

Image quantification was conducted by multiple investigators to reduce individual bias. All images were prepared using consistent brightness, contrast, and color adjustments in Imaris.

#### DNA damage foci analysis

Cell numbers and γH2AX staining patterns were quantified manually. Nuclei were identified by DAPI staining. Each nucleus was scored as γH2AX-positive if it contained at least one distinct, well-focused punctum with intensity clearly above the local nuclear background. Diffuse or ambiguous speckles were considered negative. Nuclei exhibiting uniform, whole-nucleus γH2AX staining were classified as pan-nuclear. The normalized levels of γH2AX foci or pan-nuclear staining were expressed as the ratios of the number of γH2AX-positive or pan-nuclear nuclei to the total number of nuclei scored per embryo.

#### Transmission electron microscopy

Embryos were immersed in a fixative solution (2.5% glutaraldehyde buffered in 0.1 M phosphate buffer, pH 7.4) for 1 hour at RT and stored at 4°C. Fixed samples were then washed in 0.1 M phosphate buffer, followed by post-fixation in 2% osmium tetroxide in 0.1 M phosphate buffer, pH 7.4, at 4°C for 2 hours. Subsequently, embryos were dehydrated in ethanol, followed by acetone, and then resin infiltrated and embedded in LX-112 (Ladd Research). Ultrathin sections (∼80-100 nm) were prepared using an Ultramicrotome EM UC7 (Leica) and transferred onto Formvar-stabilized slot grids. Sections were then contrasted with uranyl acetate followed by lead citrate. The slot grids were examined in a HT7700 transmission electron microscope (Hitachi High-Technologies,) at 80 kV, and digital images were acquired using a 2k × 2k Veleta CCD camera (Olympus Soft Imaging Solutions) at the Electron Microscopy Core Facility (EMiI) at Karolinska Institutet.

Ultrastructural assessment of mitochondrial and cytoplasmic lattices integrity was performed. Mitochondrial structure was analyzed qualitatively by assessing shape, signs of vacuolization, swelling, membrane integrity, and cristae maturity (poorly developed versus well-developed). Cytoplasmic lattices were examined qualitatively for fiber integrity and quantitatively for fiber abundance. For fiber abundance, a 4 × 4 μm region of interest (ROI) was selected within the cytoplasm, avoiding organelles that could obscure the lattices. This fixed ROI size ensured consistency across all analyzed images while being large enough to capture a sufficient density of cytoplasmic lattice fibers. Although the ROI dimensions remained constant, its placement was adjusted in each image to focus on cytoplasmic lattices while excluding irrelevant structures. Only images captured at 2 μm magnification were analyzed, as this magnification provided optimal clarity, making cytoplasmic lattice fibers easily distinguishable and countable. Additionally, multiple images from different regions of the embryo were analyzed at each developmental stage to account for potential spatial variations in cytoplasmic lattices distribution.

### Quantification and statistical analysis

GraphPad Prism 10 software (GraphPad Software) was used for statistical analysis. To determine the use of the parametric tests or the non-parametric tests, normality and homogeneity of variances were assessed by Shapiro-Wilk test. Since data did not assume normal distribution, comparisons between fresh and vitrified groups were performed using a two-tailed Mann-Whitney test. Statistical significance was defined as follows: ns, *p* > 0.05; ∗*p* < 0.05; ∗∗*p* < 0.01; ∗∗∗*p* < 0.001; ∗∗∗∗*p* < 0.0001. Results are presented as mean ± SD for bar graphs, and as median with interquartile range, with whiskers indicating minimum and maximum values, for box plots. All data are derived from at least two independent experiments. The exact number of embryos analyzed per condition is indicated in the corresponding figure legends.

### Additional resources

This study did not generate any additional resources.
