# Supplementary material for: Vitrification-warming delays preimplantation development and impairs mitochondrial function and cytoplasmic lattices integrity in mouse embryos
Source: iScience. 2026 Apr 22;29(6):115859. doi: 10.1016/j.isci.2026.115859 (PMC13186011; doi:10.1016/j.isci.2026.115859)
Supplement: Document S1. Figures S1–S5 [file mmc1.pdf]

iScience, Volume *29*

Supplemental information

Vitrification-warming delays preimplantation development and impairs mitochondrial function and cytoplasmic lattices integrity in mouse embryos

Mariana T. Barroso, Jose A. Rodriguez Muñoz, Viola Sjöström, Konstantina Dindini, Jian Zhao, Kenny A. Rodriguez-Wallberg, and Arturo Reyes Palomares

Supplementary materials:

- Figures S1-S5

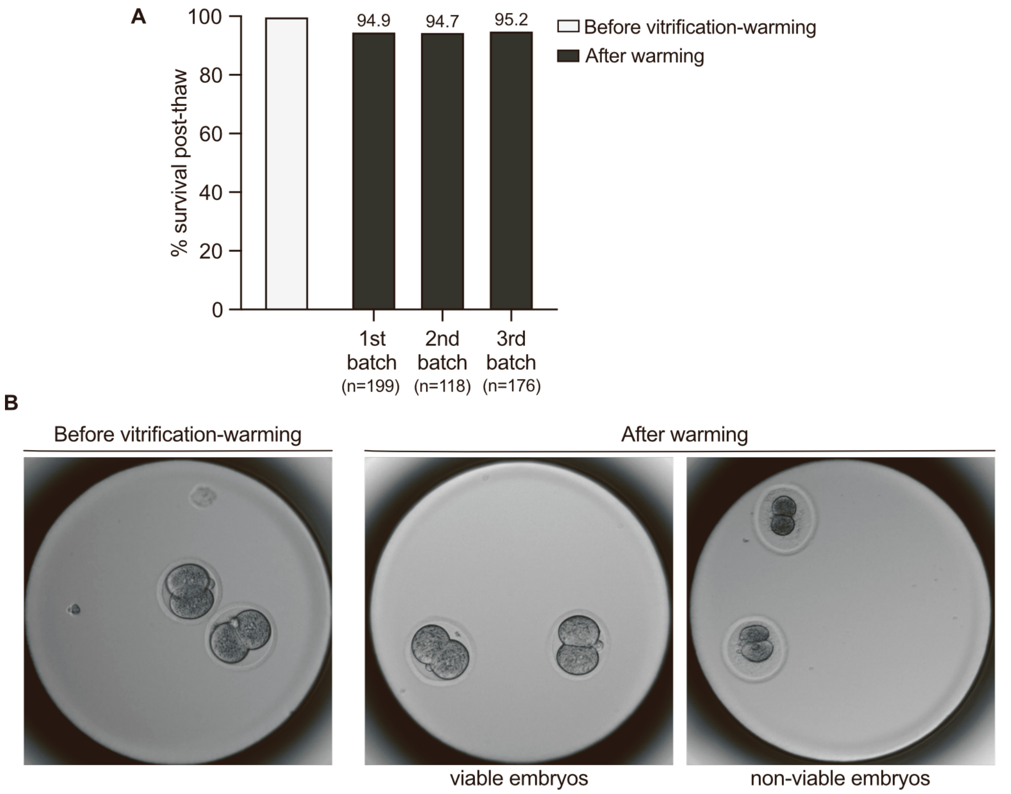

Figure S1. Survival rate of 2-cell stage mouse embryos after warming, related to Figure 1. (A) Survival rate of three independent mouse embryo batches immediately after warming (dark columns). The first column (100% survival) corresponds to embryos before vitrification-warming and serves as the baseline control. (B) Representative bright-field images acquired in the Geri® incubator at 10× magnification, showing 2-cell stage embryos before and after vitrification-warming. Images of both viable (showing successful re-expansion shortly and intact blastomeres) and non-viable embryos (failed re-expansion) are shown for comparison.

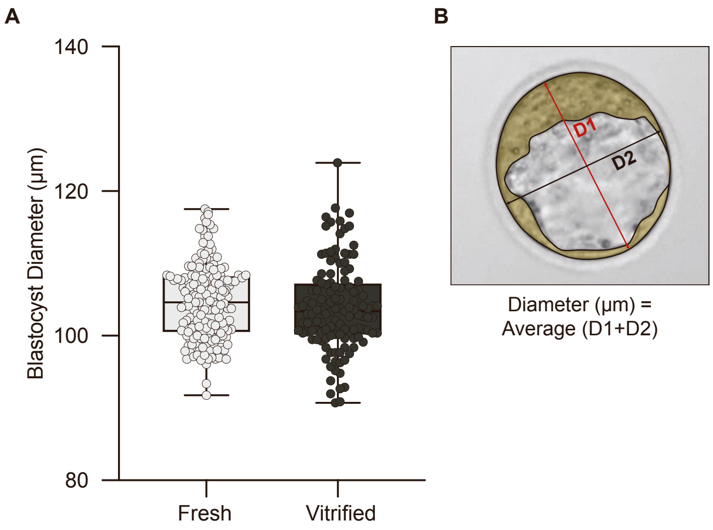

Figure S2. Measurement of blastocyst diameter (µm) between fresh and vitrified groups, related to Figure 1. (A) Graphical representation of the diameters of fresh (N=229) and vitrified (N=212) blastocysts. (B) Schematic representation of the diameter measurement. The blastocyst diameter was calculated by averaging two cross-sectional measurements (D1 + D2), excluding zona pellucida. Measurements were performed on time-lapse microscopy images at the time each embryo reached fully expanded blastocyst stage.

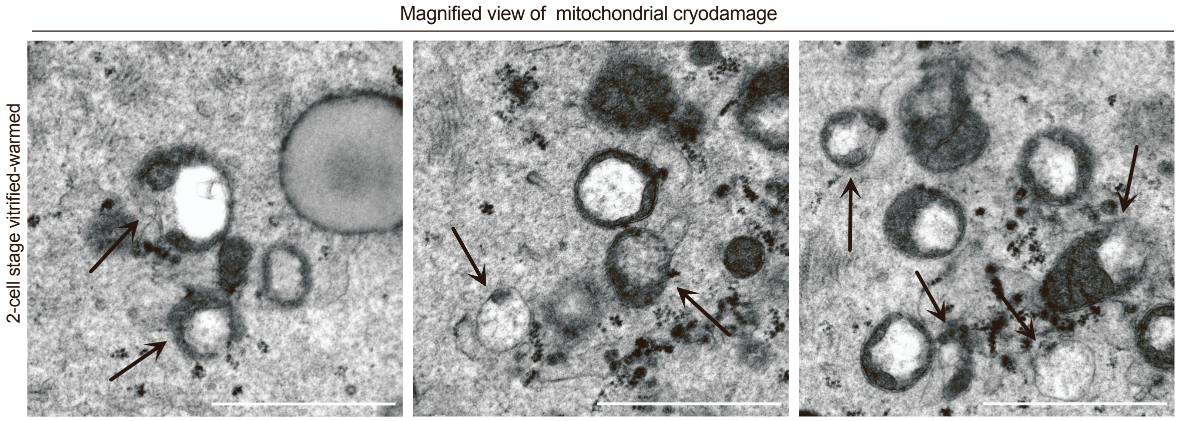

Figure S3. Mitochondrial membrane damage in vitrified 2-cell stage embryos, related to Figure 3. Representative magnified transmission electron micrographs showing mitochondrial ultrastructure in 2-cell stage embryos following vitrification and warming. All micrographs are cropped regions of interest obtained from a larger stitched TEM image acquired at 15,000x magnification. Black arrows indicate damaged mitochondria, characterized by disrupted membranes. Scale bars represent 1μm.

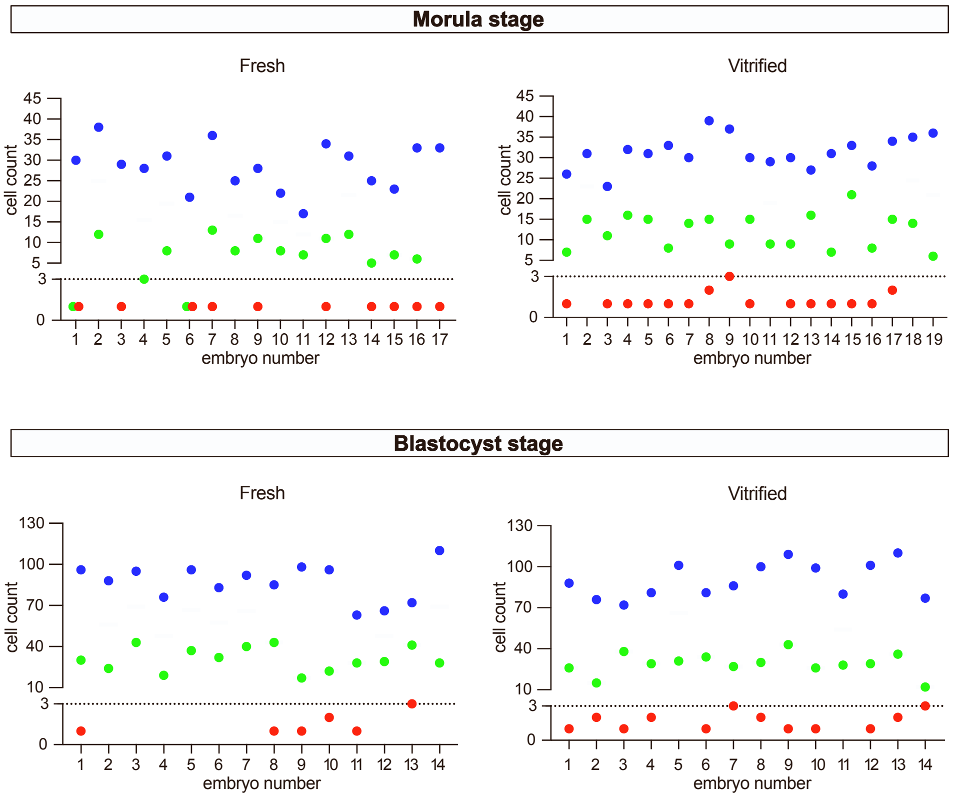

Figure S4. Per-embryo absolute counts of blastomeres, bright foci, and pan-nuclear signal, related to Figure 7. Graphs show per-embryo absolute counts of total blastomeres, blastomeres with bright foci, and blastomeres with pan-nuclear signals in morula and blastocyst embryos, fresh or vitrified. For each embryo (x-axis), blue dots indicate total blastomeres (DAPI), green dots indicate bright foci, and red dots indicate pan-nuclear signals. The y-axis is broken to show 0–3 and higher values, allowing visualization of pan-nuclear counts alongside larger DAPI and bright foci counts.

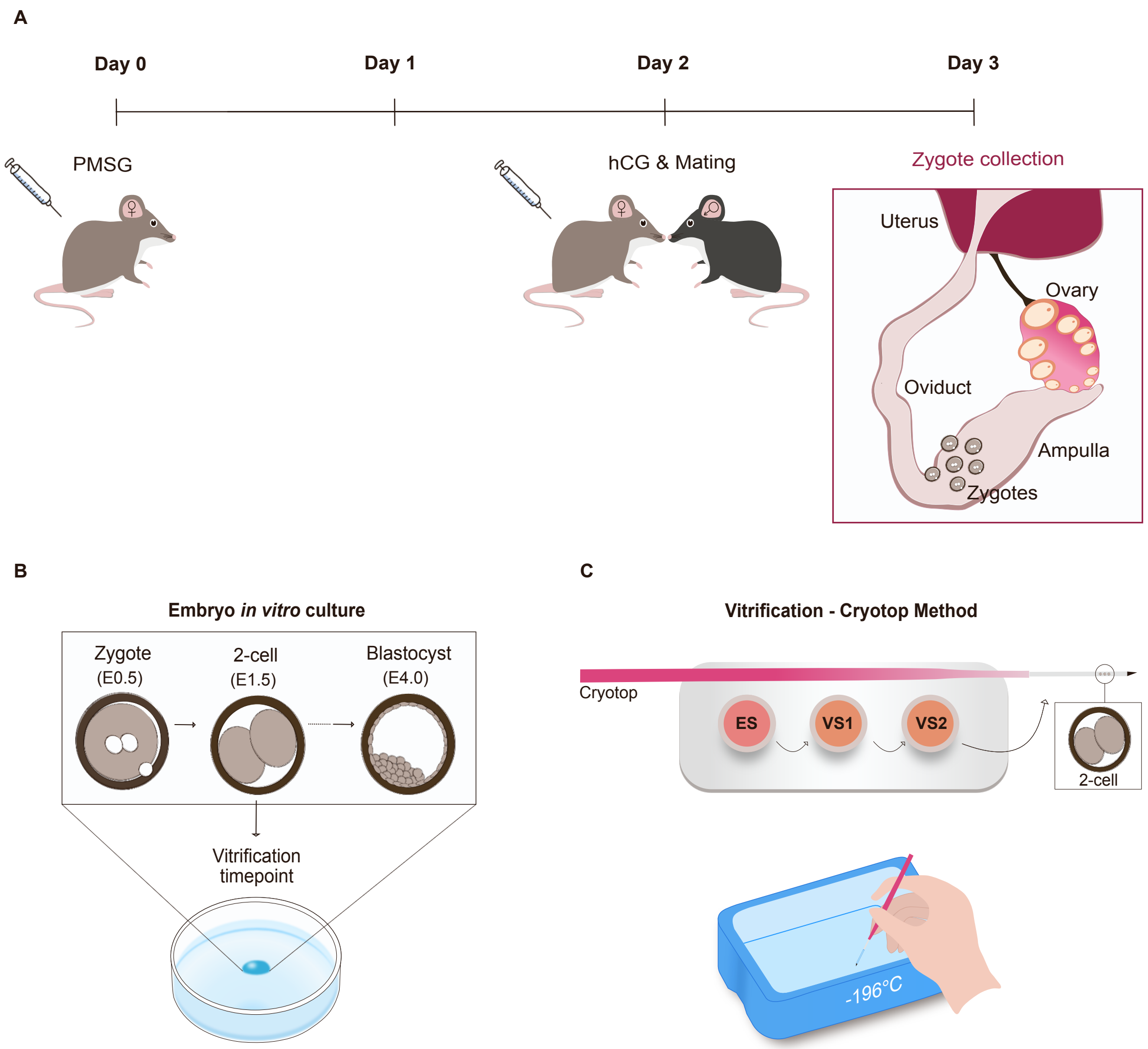

Figure S5. Experimental setup, related to STAR Methods. (A) Schematic timeline of mice superovulation, mating and zygote collection. Superovulation was induced with pregnant mare serum gonadotropin (PMSG) on Day 0, followed by human chorionic gonadotropin (hCG) on Day 2. Following hCG injection, females were mated with males. Zygotes were collected from the ampulla on Day 3 after dissection of the ovaries and oviducts. **(B)** Zygotes (E0.5) were cultured *in vitro* to the blastocyst stage (E4.0). Embryos were vitrified and warmed at the 2-cell stage (E1.5), as indicated. **(C)** Schematic representation of the vitrification procedure using the Cryotop method. Embryos were first incubated in Equilibration Solution (ES) for gradual dehydration and initial cryoprotectant exposure. Embryos were then sequentially exposed to Vitrification Solution 1 (VS1) and Vitrification Solution 2 (VS2), which contain increasing concentrations of cryoprotectants. Embryos were then loaded onto a Cryotop strip and immediately submerged in liquid nitrogen (–196 °C). Affinity Designer was used to create the illustration.
